# Saccharin Aggravates Eosinophilic Esophagitis via MAPK3 Interaction: A Network Toxicology and Machine Learning Study With SPR Analysis

**DOI:** 10.1002/fsn3.71409

**Published:** 2026-01-07

**Authors:** Yuan Yang, Tao Guo, Peiyuan Li, Kangle Gao, Xufeng Ning, Lingshan Zhou, Weiwei Zhou, Bin Zeng

**Affiliations:** ^1^ Department of Gastroenterology The First Affiliated Hospital, Hengyang Medical School, University of South China Hengyang China; ^2^ Department of Geriatrics Ward 2 The First Hospital of Lanzhou University Lanzhou China

**Keywords:** eosinophilic esophagitis, machine learning, MAPK3, network toxicology, saccharin, SPR analysis

## Abstract

The potential role of artificial sweeteners in eosinophilic esophagitis (EoE) remains poorly understood. This study aimed to investigate the molecular mechanism by which saccharin might exacerbate EoE. We integrated network toxicology with machine learning approaches to identify core pathogenic genes of EoE. The interactions between saccharin and the predicted targets were validated via molecular docking, molecular dynamics (MD) simulations, and surface plasmon resonance (SPR). Our analysis identified MAPK3, CPS1, and HS3ST1 as potential EoE‐related targets of saccharin. Molecular docking demonstrated strong binding affinities between saccharin and these proteins, which was confirmed by stable binding via molecular dynamics simulations. Further SPR analysis revealed that saccharin binds directly to MAPK3. This study demonstrated that saccharin potentially aggravates EoE by directly targeting MAPK3 to activate pro‐inflammatory pathways, highlighting a novel dietary risk factor and underscoring the need for a safe reevaluation for susceptible populations.

## Graphical abstract

This study provides a novel molecular perspective on the role of dietary factors in EoE pathogenesis. We establish saccharin as a key aggravating factor for EoE and highlight MAPK3 as a compelling therapeutic target. Our findings suggest that controlling saccharin intake represents a direct and beneficial dietary management strategy for EoE. We offer crucial evidence for re‐evaluating the safety of saccharin in susceptible populations, bridging food additive exposure to EoE risk.

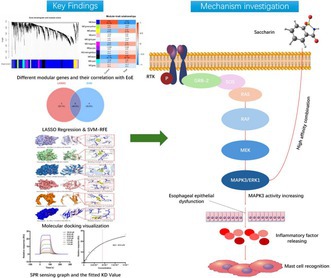

## 1 Introduction

EoE is a chronic, Th2‐related inflammatory disease characterized by eosinophil‐predominant infiltration of the esophageal epithelium (Ray et al. 2024). The pathophysiology involves multifactorial interactions, including genetic susceptibility (Martin et al. 2018), allergic responses (McGowan et al. 2025), microbial dysbiosis (D'Souza et al. 2021), epithelial barrier impairment (Braskett et al. 2025), and tissue remodeling (Kennedy et al. 2025). In recent decades, the global prevalence of EoE has increased dramatically, with the worldwide burden increasing persistently (Hahn et al. 2023). This epidemiological surge likely stems from modern lifestyle shifts, particularly dietary pattern changes such as the increasing consumption of processed foods and preservatives in Westernized diets (Farah et al. 2025). In addition to traditional allergens, artificial additives can also lead to allergic reactions by influencing the body's immune functions (Aldabayan 2025). Previous studies have confirmed that synthetic additives can disrupt esophageal immune homeostasis. For example, food additives can act as allergens to trigger IgE‐mediated immune reactions or as pseudoallergens to trigger non‐IgE‐mediated immune reactions, thereby causing esophageal inflammation (Velázquez‐Sámano et al. 2019). This suggests that the widespread consumption of artificial food additives might be a contributing factor to the epidemiologic rise in this condition.

Artificial sweeteners (AS), especially saccharin, are widely used in low‐calorie products because of their high sweetness intensity and minimal impact on blood glucose (Sylvetsky and Rother 2016). Although AS provide sweetness without the caloric load of sugar and are generally considered nontoxic at recommended doses in the short term (Al‐Ishaq et al. 2023), emerging evidence indicates that they possess unexpected immunomodulatory (Zani et al. 2023) and pro‐inflammatory properties (Wu et al. 2025). For example, studies have shown that long‐term or high‐dose saccharin intake sustains oxidative stress pathways, inducing chronic inflammation and causing DNA damage. It has also been associated with hepatorenal toxicity and abnormal proliferation of the bladder epithelium, suggesting possible carcinogenic effects (Shi et al. 2021; Azeez et al. 2019). Several studies have demonstrated that artificial sweeteners can disrupt the gut microbiota balance (Guo et al. 2021; Shil and Chichger 2021). Gut dysbiosis is increasingly recognized as a factor influencing systemic and mucosal immune responses (Suez et al. 2014). AS may also contribute to modulating immune cell functions, ultimately affecting the body's immune system (Ma et al. 2024; Yu et al. 2023; Künzel et al. 2024). For example, saccharin sodium impairs immune regulation by hindering antigen‐presenting cell (APC) migration and reducing regulatory Treg proportions, thereby promoting allergic reactions (Yamashita et al. 2017). The gastrointestinal tract and the esophagus share commonalities in mucosal immunity (Rossi et al. 2022). Given these shared immunological features, these findings suggest that saccharin might directly interact with EoE‐associated inflammatory proteins, thereby exacerbating disease progression. However, direct evidence exploring the molecular link between saccharin exposure and esophageal‐specific inflammation in EoE is still lacking. There is a critical need to elucidate whether and how saccharin acts as a potential dietary risk factor in EoE pathogenesis.

To investigate the role of saccharin in EoE, we employed an integrated strategy. Starting with network toxicology to identify key targets, we progressed through molecular docking and dynamics simulations, ultimately confirming direct binding between saccharin and MAPK3 via SPR. This binding event provides a mechanistic basis for how saccharin may activate the MAPK/ERK pathway and exacerbate EoE.

## 2 Materials and Methods

### 2.1 Data Acquisition

The transcriptomic dataset GSE228083 related to EoE was downloaded from the Gene Expression Omnibus (GEO) database (https://www.ncbi.nlm.nih.gov/), comprising esophageal mucosal biopsy samples from 12 normal controls and 12 EoE patients.

### 2.2 Weighted Gene Co‐Expression Network Analysis (WGCNA)

WGCNA was performed on the gene expression matrix obtained from GEO. The soft‐thresholding power was set to 6 to achieve a scale‐free co‐expression network, which maintained moderate mean connectivity. Prior to analysis, genes exhibiting low expression levels and variance were filtered out to minimize background noise. Dynamic tree cutting was then employed to identify expression modules, and a minimum module size of 50 genes was set. Modules with highly correlated eigengenes were merged, resulting in a total of 20 distinct co‐expression modules. The module eigengenes (MEs) were calculated. The key module most significantly associated with EoE pathogenesis was identified by correlating MEs with clinical traits. All genes within this key module were extracted for subsequent analysis.

### 2.3 Screening for Overlapping Targets Between EoE and Saccharin

Predictions from the Comparative Toxicogenomics Database (CTD; https://ctdbase.org/) (accessed: June, 2025) and SwissTargetPrediction (http://swisstargetprediction.ch/) (accessed: June, 2025) were integrated to identify potential targets of saccharin, and duplicates were removed from the resulting dataset. The candidate gene set emerged from intersecting key module genes identified via WGCNA with potential saccharin targets.

### 2.4 Identification of Core Genes via LASSO Regression and SVM‐RFE

LASSO regression with ten‐fold cross‐validation was performed using the glmnet package to optimize lambda selection. This step helps identify key EoE‐related feature genes from the candidate pool. Subsequently, SVM‐RFE was applied for filtering to further refine the key feature genes. Finally, the intersecting genes derived from both the LASSO and SVM‐RFE results were selected as the potential core genes through which saccharin may promote EoE pathogenesis.

### 2.5 Molecular Docking

On the basis of screening‐identified core genes, we selected proteins displaying marked expression variations that are closely connected to EoE pathogenesis. Their high‐definition crystal structures were obtained from the Protein Data Bank (PDB). Next, saccharin's molecular structure was sourced from the PubChem database and underwent conversion followed by energy minimization via Open Babel. Molecular docking was conducted with AutoDock Vina 1.2.5 to determine the most favorable binding mode, precise binding sites, and corresponding binding energies for saccharin‐core protein complexes. The protein structures demonstrating the highest binding affinities were then rendered and visualized via PyMOL.

### 2.6 Molecular Dynamics Simulation

The optimal saccharin‐core protein complex derived from docking served as the initial conformation and was immersed in an explicit solvent box with counterions added to neutralize the system charge (Table 1). The ff14SB force field from AMBER was used to parameterize the complex alongside the TIP3P water model filling the solvent box, and the simulation system ensured that the water box edge resided no less than 12 Å from the complex's outermost atoms. Following system construction, energy minimization proceeded via GROMACS and AmberTools, followed by NVT/NPT equilibration to stabilize the system. Production simulations were then conducted under physiological conditions (300 K, 1 atm) for ≥ 100 ns, with temperature, pressure, and other parameters correctly configured while trajectories were recorded. This simulation time scale was selected because it is well established in comparable studies for protein‐ligand complexes. Besides, we ensured sufficient time for the system to reach equilibrium and for key stability metrics to converge, thereby providing a reliable assessment of binding stability and affinity (Zeng et al. 2025; Zhou et al. 2025). System stability (RMSD), residue flexibility (RMSF), radius of gyration (Rg), hydrogen‐bond counts, and binding free energies (employing MM/PBSA or MM/GBSA) were calculated to evaluate thermodynamic stability, binding affinity, and key interacting residues.

**TABLE 1:** Docking grid box parameters for receptor proteins.

Protein	PDB/ID	Center (X, Y, Z)	Size (X × Y × Z)
CPS1	6UEL	72, 56, 32	24 × 24 × 24
HS3ST1	1ZRH	12, 25, 14	24 × 24 × 24
HS3ST4	Q9Y661	1, −7, −3	24 × 24 × 24
IL1RL1	4KC3	46, −28, 12	24 × 24 × 24
MAPK3	4QTB	34, 59, 53	21 × 15 × 17
WNT3	6AHY	7, −8, −34	49 × 43 × 54

### 2.7 SPR Validation

In this study, the core protein was immobilized on the CM5 sensor chip surface via amine coupling chemistry. Specifically, 10 mM sodium acetate buffer (pH 5.5) was used as the coupling buffer, with a flow rate of 20 μL/min and a protein concentration of 20 μg/mL. These conditions were optimized through preliminary experiments to achieve a stable immobilization level of approximately 12,000 response units (RU) while preserving protein activity, thereby ensuring the reliability of subsequent binding assays.

Saccharin solutions were prepared from a stock solution in 1% DMSO (catalog no. D8418, Sigma, USA) and subsequently diluted to the required concentrations with 10 × PBS‐P+ (catalog no. 28995084, Cytiva, USA) for SPR analysis. The core protein was immobilized onto a CM5 sensor chip (catalog no. BR‐1005‐30, Cytiva, USA) surface using Biacore T200 and an amino coupling kit (catalog no. BR100050, Cytiva, USA). Throughout the immobilization process, the instrument's operational protocols were followed to ensure the protein was securely and uniformly attached. During the immobilization step, 10 mM sodium acetate buffer (catalog no. BR100349, Cytiva, USA) was employed as the coupling buffer. For kinetic analysis, the running buffer was PBST (10 mM PBS, 0.05% Tween‐20, pH 7.4) containing 1% DMSO to maintain saccharin solubility. Background signals were subtracted via both blank (running buffer) injections and a reference flow cell without immobilized protein to correct for bulk refractive index changes and nonspecific binding to the sensor chip matrix.

Saccharin solutions, prepared at various concentrations, were subsequently injected as analytes across the protein‐modified chip surface. The flow rate and contact time were precisely calibrated to guarantee adequate interaction duration between the analyte molecules and the immobilized protein. The association rate constant (*ka*), dissociation rate constant (*kd*), and equilibrium dissociation constant (*KD*) precisely characterize the binding affinity and kinetic profile of saccharin interactions with the core protein. This validation unequivocally establishes the robustness of the binding mechanism between saccharin and the core protein. All reagents used were of analytical grade or higher and were used within their recommended shelf life.

## 3 Results

### 3.1 WGCNA Analysis

**FIGURE 1:**
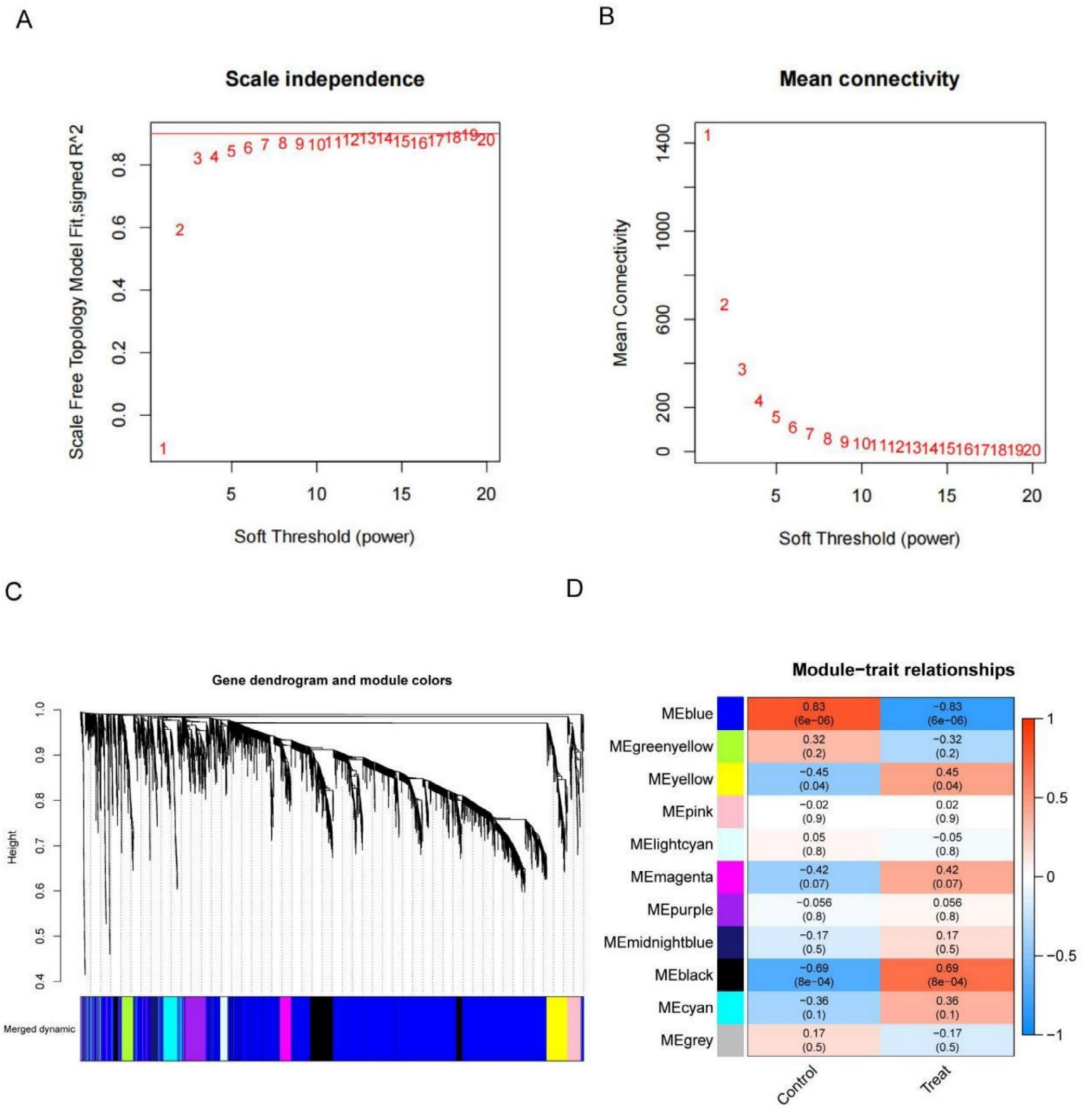
(A) Scale independence analysis showing the *R* ^2^ values for different soft threshold powers. (B) Mean connectivity as a function of the soft threshold power. Gene dendrograms and module colors from WGCNA. (C) The dendrogram illustrates the hierarchical clustering of genes, and the colors represent different gene modules. (D) Module‐trait relationships. The heatmap illustrates the correlation between different modules and EoE traits.

In this study, WGCNA was utilized to identify gene modules associated with EoE. As shown in Figure 1A, this value achieved a scale‐free topology model fit of over 0.8, indicating an appropriate balance between biological relevance and network connectivity and maintaining moderate mean connectivity (Figure 1B). Using this parameter, we constructed a gene dendrogram (Figure 1C), which classified genes into different modules through a dynamic tree‐cutting method, with each module identified by a unique color. The heatmap in Figure 1D shows the correlation between modules and EoE traits, where the MEblue module exhibited a significant negative correlation with EoE (*r* = −0.83, *p* < 0.05), whereas the MEblack module showed a positive correlation (*r* = 0.69, *p* < 0.05).

### 3.2 Core Gene Screening

**FIGURE 2:**
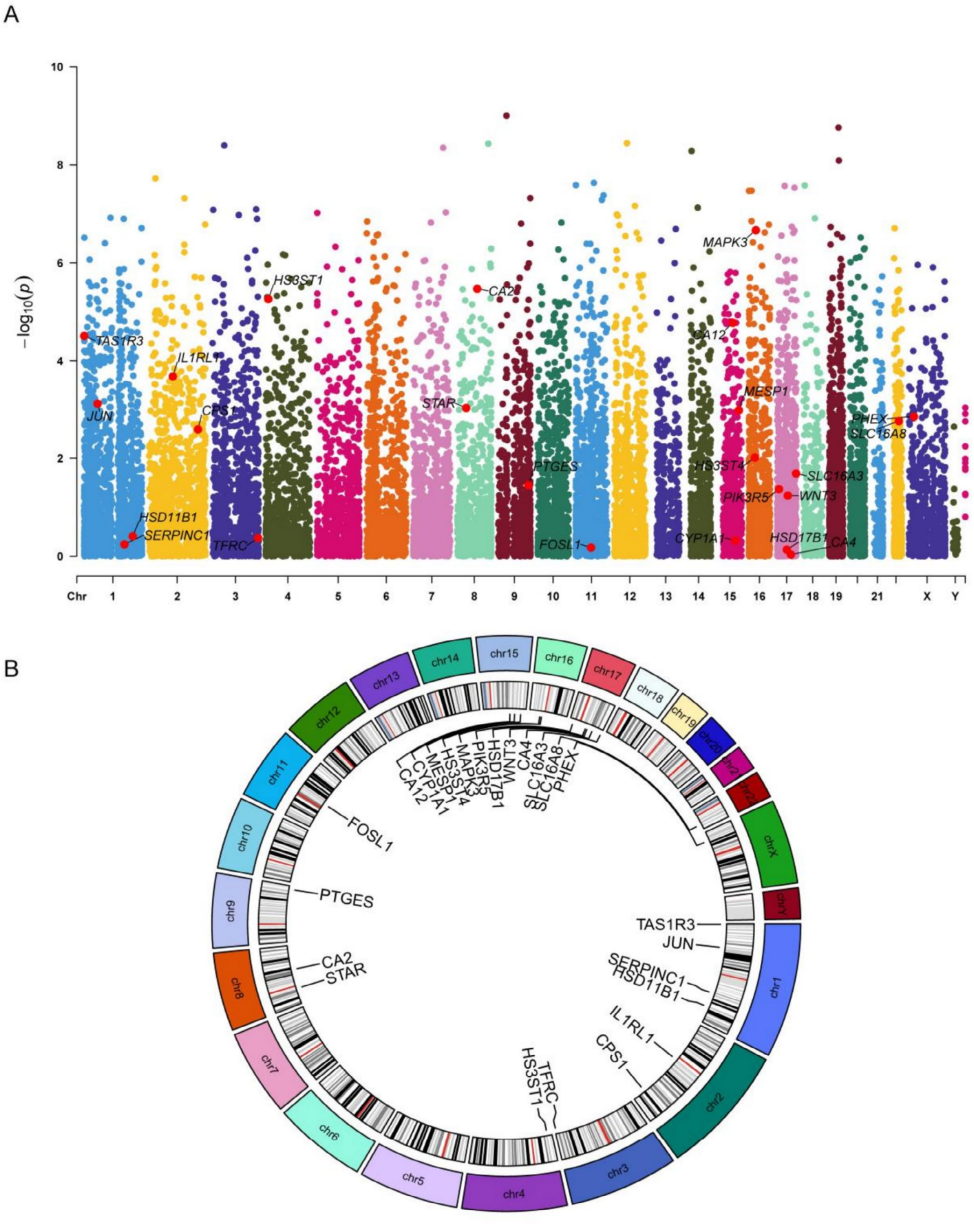
Core gene selection and co‐expression network analysis: (A) Manhattan plot showing the significance levels of genes across various chromosomes, as indicated by the −log_10_ (*p* value). (B) Gene co‐expression network diagram.

Eighty‐eight potential saccharin targets were predicted through integration of multiple databases. Next, we intersected the key module genes identified through WGCNA with these potential targets, yielding a focused shortlist of 24 candidate genes. To pinpoint the core genes, LASSO regression and SVM‐RFE algorithms were employed. Our initial analysis utilized a Manhattan plot to reveal gene significance levels across different chromosomes. Genes such as MAPK3 and HS3ST1, marked in red, demonstrated striking significance, suggesting a strong association with EoE (Figure 2A). The circular gene co‐expression network diagram in Figure 2B clearly depicts the gene distribution and co‐expression relationships across chromosomes. Marked genes, including HS3ST4, WNT3, and MAPK3, occupied pivotal positions within this network, underscoring their potential key roles in regulatory networks.

**FIGURE 3:**
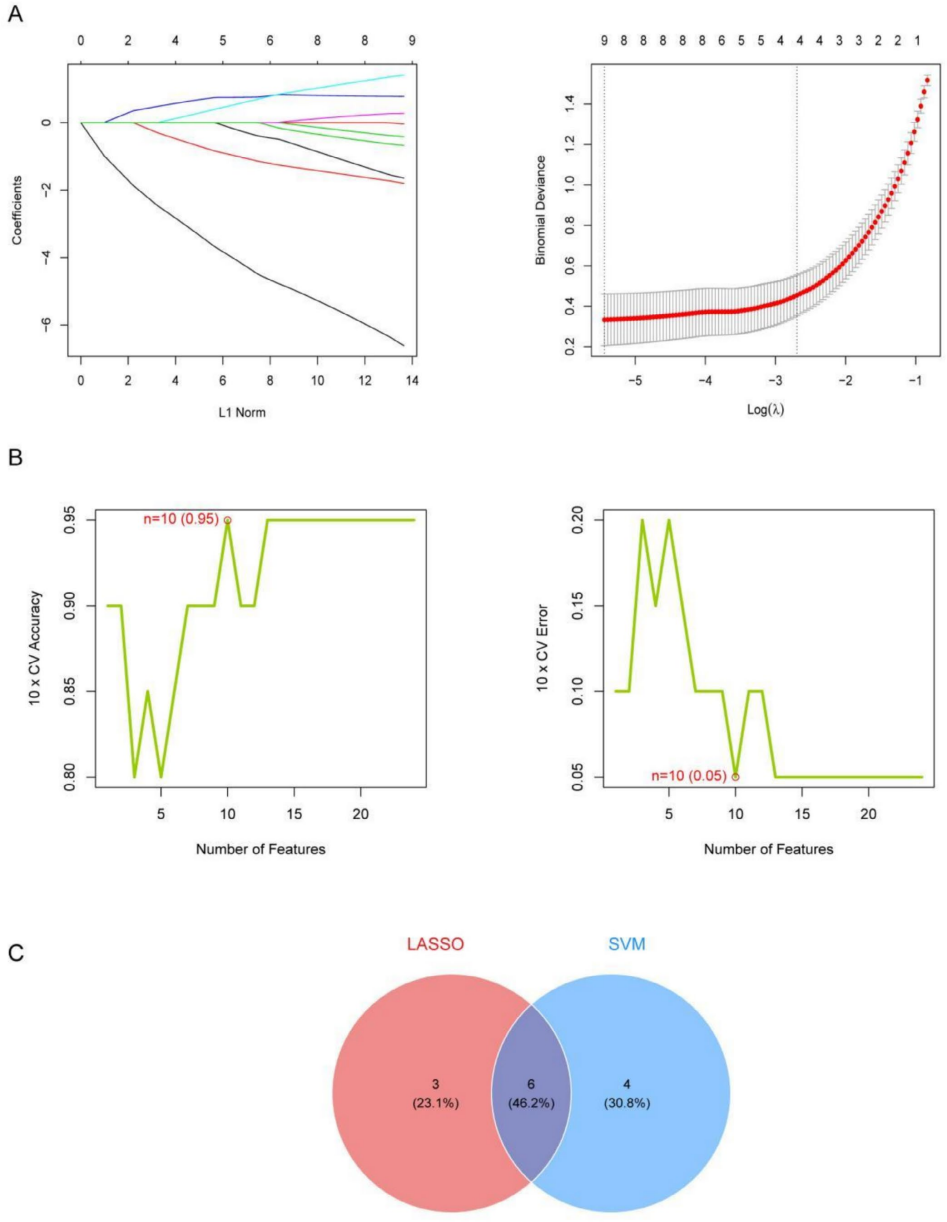
Core gene selection: (A) LASSO regression analysis graph: The left panel shows the variation in model coefficients under different lambda values, whereas the right panel presents the bias analysis for LASSO regression. (B) Cross‐validation analysis graph. (C) Venn diagram: The intersection and differences among the core genes identified by both the LASSO and SVM‐RFE methods are shown.

During the core gene screening phase, we employed the LASSO regression analysis depicted in Figure 3A to determine the optimal lambda value, effectively preventing model overfitting. The optimal number of features is 9. Cross‐validation analysis in Figure 3B demonstrated that the SVM‐RFE regression model achieved its best performance with 10 features, with a robust cross‐validation accuracy of 0.95 while the error rate plummeted to a mere 0.05.

Finally, the Venn diagram in Figure 3C revealed the intersection and distinctions among core genes selected by both the LASSO and SVM‐RFE methods, consistently pinpointing six genes common to both approaches. These six core genes—CPS1, HS3ST1, HS3ST4, IL1RL1, MAPK3, and WNT3—occupy central positions within the gene co‐expression network and demonstrate significant importance in both machine learning algorithms.

### 3.3 Molecular Docking and Molecular Dynamics Simulation

**FIGURE 4:**
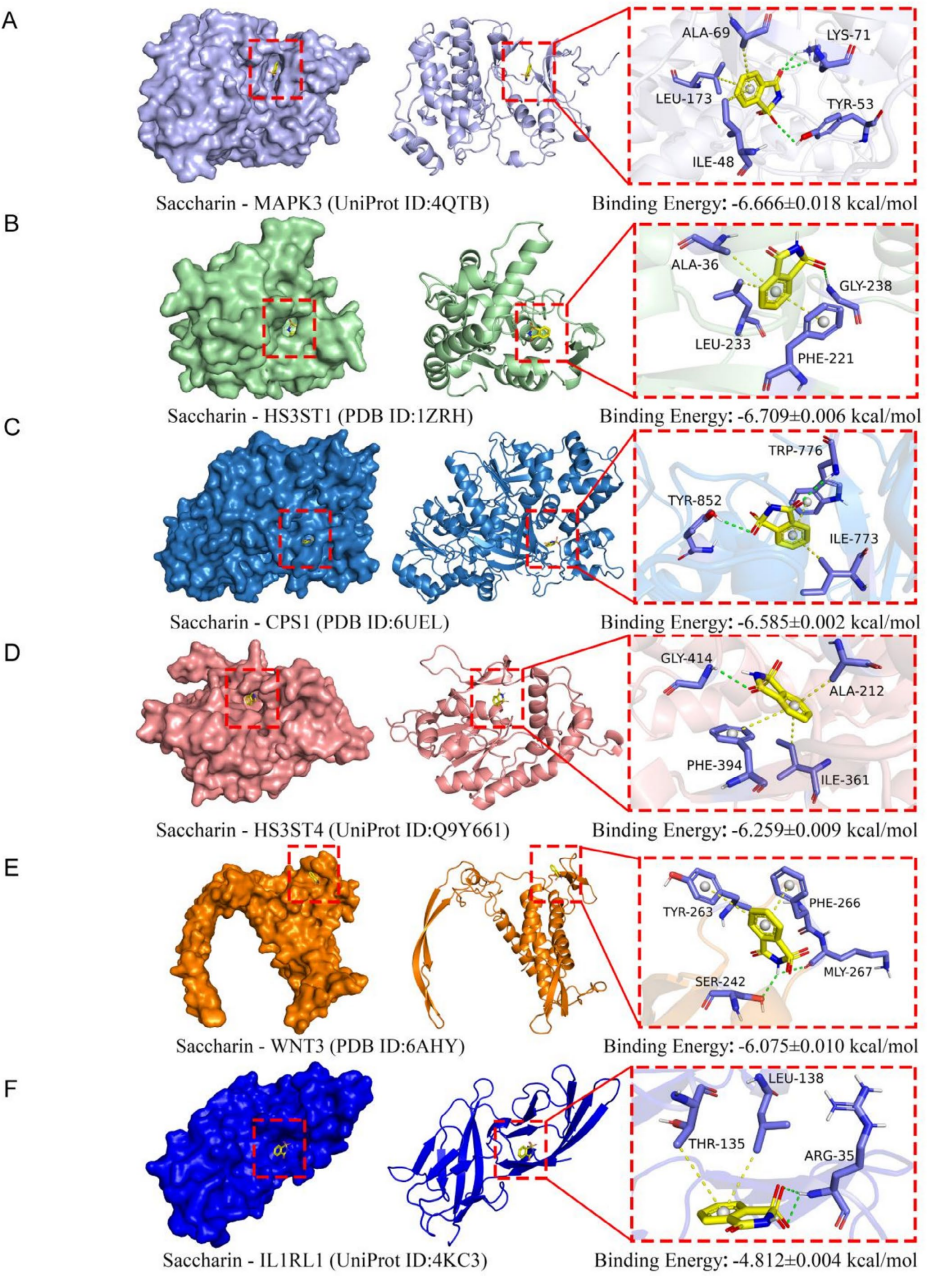
Molecular docking results of saccharin with key proteins (MAPK3, HS3ST1, CPS1, HS3ST4, WNT3, and IL1RL1).

The molecular docking results are shown in Figure 4. Saccharin forms multiple binding interactions with each of the five proteins, indicating strong binding that could affect their structural functions and biological activities. The binding energy of saccharin with CPS1, HS3ST1, HS3ST4, MAPK3, and WNT3 is less than −5 kcal/mol, suggesting a high binding affinity between saccharin and these proteins. Three independent simulations were performed for each complex.

To further verify the stability and reliability of the binding between saccharin and six key proteins (CPS1, HS3ST1, HS3ST4, IL1RL1, MAPK3, and WNT3), three independent 100 ns molecular dynamics simulations were performed in this study. All three simulations yielded stable results, and the detailed results are presented in Appendix S1.

**FIGURE 5:**
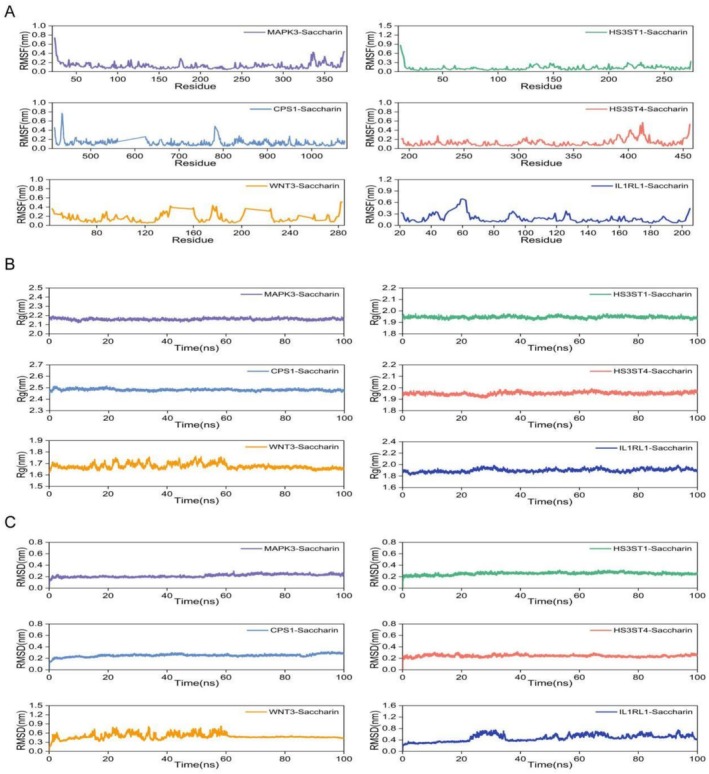
Stability of saccharin complexes with six key proteins (MAPK3, HS3ST1, CPS1, HS3ST4, WNT3, and IL1RL1): (A) RMSF curves for amino acid residues in different saccharin‐protein complexes. (B) Changes in the radius of gyration (Rg) over time for saccharin complexes with various proteins. (C) RMSD changes over time for the saccharin‐protein complexes.

**FIGURE 6:**
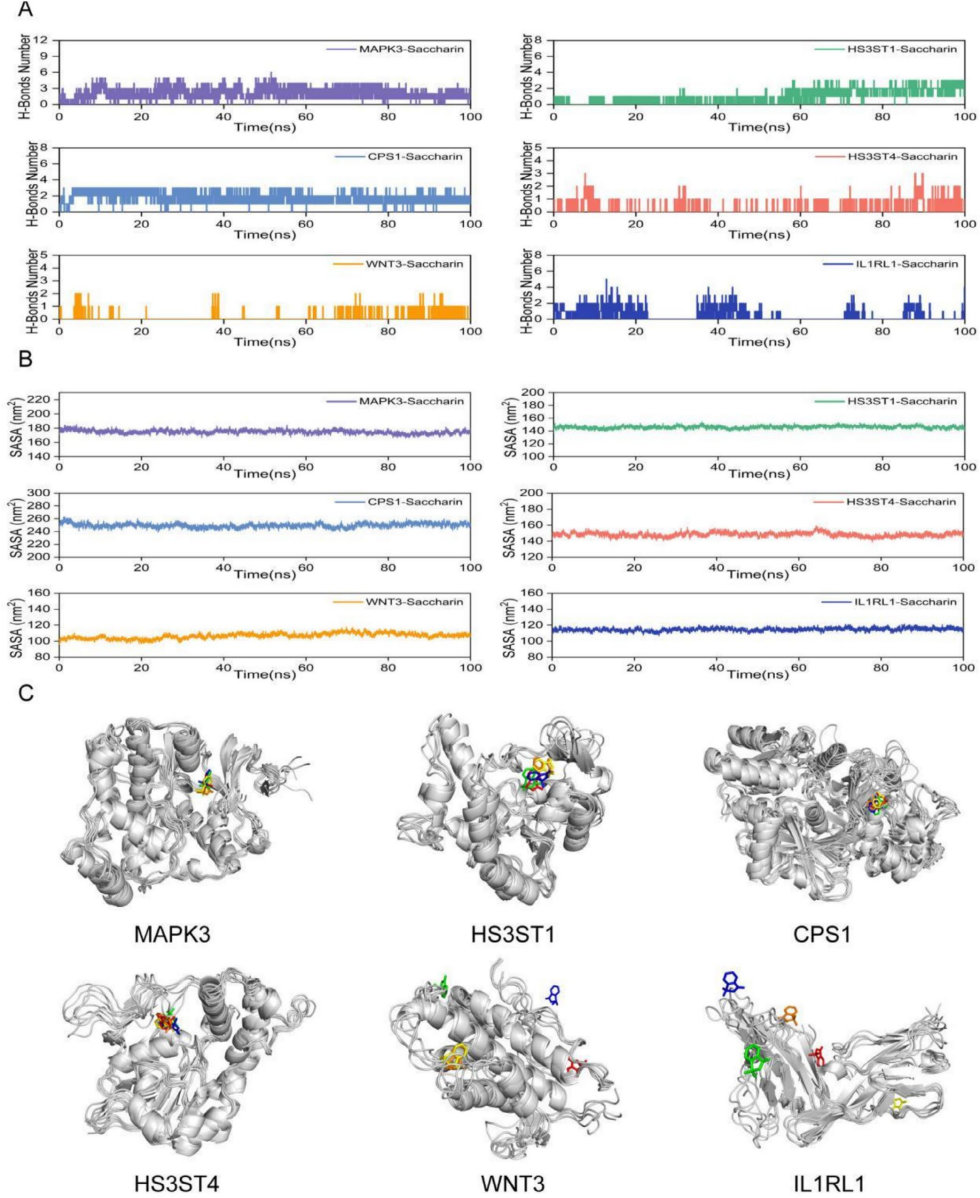
The number of (A) hydrogen bonds and (B) SASA over time for different saccharin‐protein complexes. (C) Three‐dimensional structural illustrations of each protein binding with saccharin.

As shown in Figure 5A, the RMSF analysis indicates that the fluctuations of amino acid residues in proteins CPS1, HS3ST1, HS3ST4, IL1RL1, MAPK3, and WNT3 are all within 1 nm, demonstrating that these proteins maintained high structural stability throughout the simulation. In addition, the Rg curves (Figure 5B) and SASA (Figure 6B) curves of these proteins remained stable during the simulation, further confirming that the structures of these proteins were relatively stable during the simulation period.

The RMSD analysis revealed that the complexes formed between saccharin and the proteins CPS1, HS3ST1, HS3ST4, and MAPK3 fluctuated within the range of 0.2–0.3 nm (Figure 5C), indicating that these complexes experienced minimal structural changes and exhibited high stability during the simulation. In contrast, the RMSD curves for the complexes formed with IL1RL1 and WNT3 showed greater fluctuations, suggesting lower binding stability and potential partial dissociation during the simulation.

**FIGURE 7:**
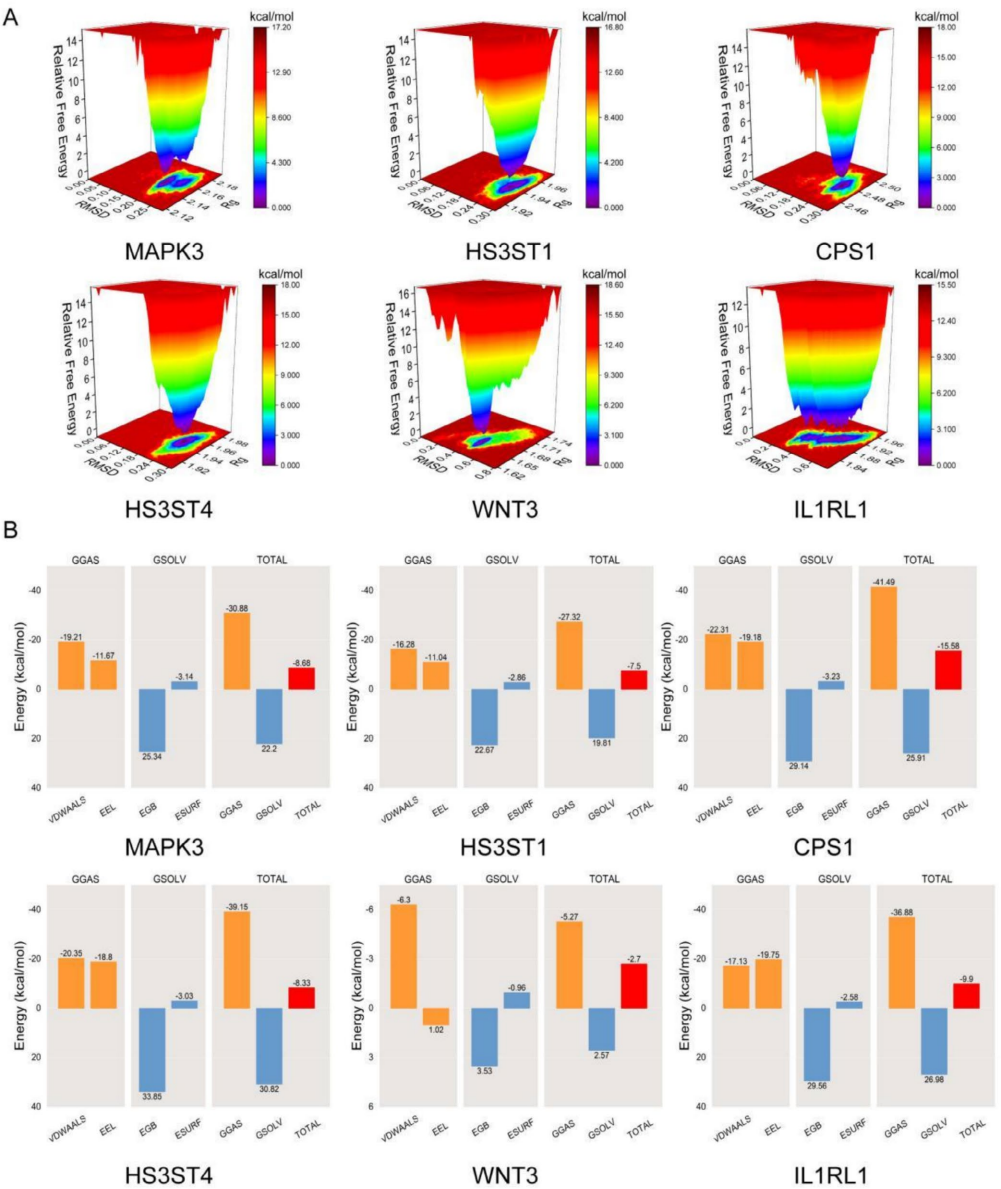
(A) Free energy landscape plots showing the distribution of binding free energy for each protein‐saccharin interaction. (B) Diagrams of average binding free energy, where VDWAALS, EEL, EGB, ESURF, GGAS, GSOLV, and TOTAL represent van der Waals forces, electrostatic energy, polar solvation energy, nonpolar solvation energy, gas phase energy, solvation energy, and total binding free energy, respectively.

Hydrogen bond analysis revealed that stable hydrogen bonds formed between saccharin and proteins CPS1, HS3ST1, HS3ST4, and MAPK3, with quantities stabilizing at approximately 2–3, 1–3, 1–2, and 2–4 bonds, respectively, further increasing the binding stability of the complexes (Figure 6A). The free energy landscape (FEL) also revealed that these complexes formed a relatively singular minimum energy cluster, indicating increased binding stability (Figure 7A). In comparison, the complexes of IL1RL1 and WNT3 with saccharin had hydrogen bonds that disappeared for extended periods during the simulation, and the FEL plots displayed multiple minimum energy clusters, suggesting that these two complexes have poorer binding stability. The calculated average binding free energy further corroborates these findings, with the average binding free energies for saccharin with proteins CPS1, HS3ST1, HS3ST4, IL1RL1, WNT3, and MAPK3 being −15.58 ± 0.18, −7.5 ± 0.14, −8.33 ± 0.1, −9.9 ± 0.21, −2.7 ± 0.51, and −8.68 ± 0.11 kcal/mol, respectively, indicating high binding affinity (Figure 7B).

### 3.4 SPR Validation

**FIGURE 8:**
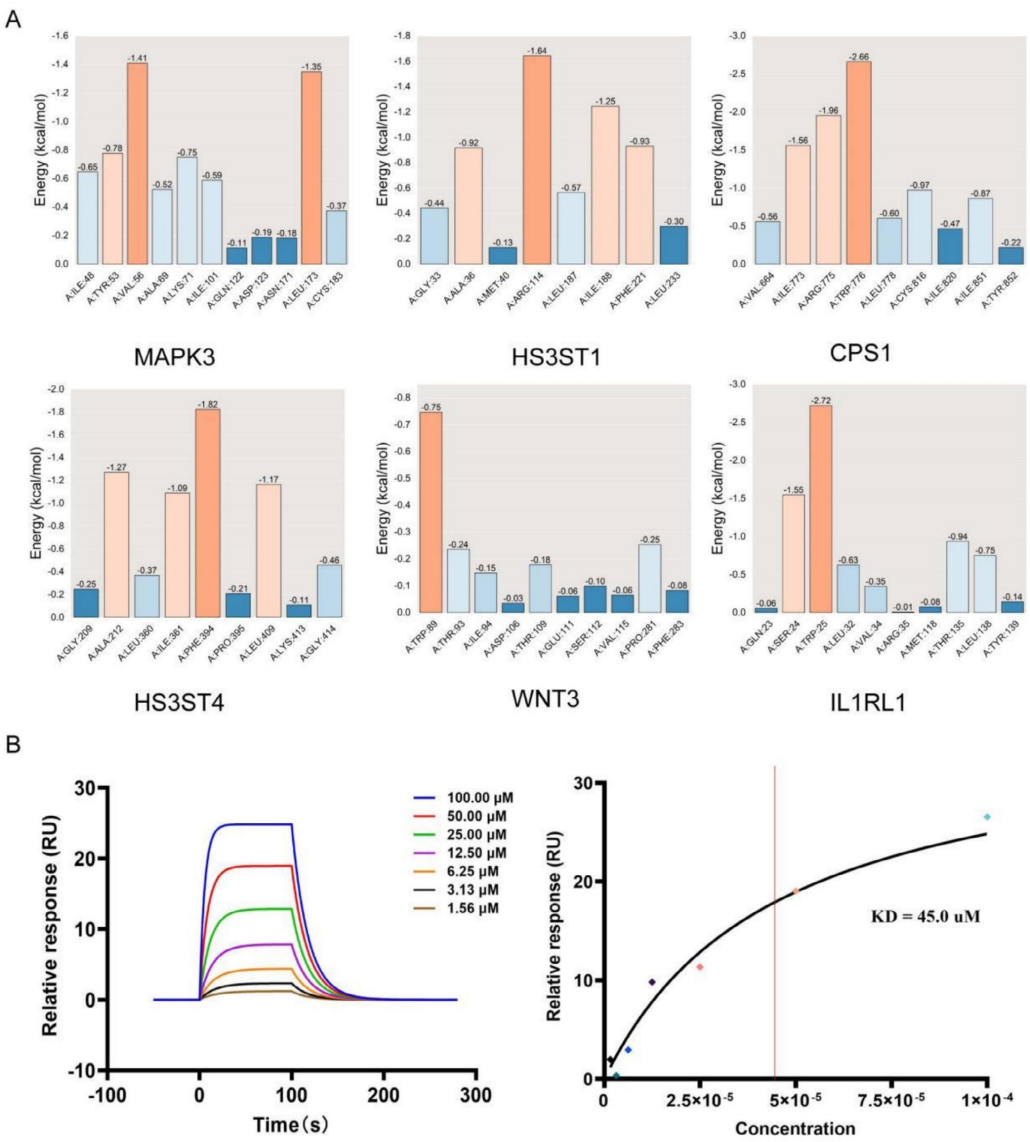
(A) Comparisonc of amino acid residue energy contributions. (B) SPR sensorgram and the fitted *KD* value.

In this study, we employed SPR technology to quantitatively evaluate the binding affinity between saccharin and the core protein. During the experiment, interactions between varying concentrations of saccharin (ranging from 1.56 to 100.00 μM) and immobilized MAPK3 on a CM5 chip were observed. The concentration range was determined on the basis of preliminary experiments to ensure appropriate SPR response signal intensity, avoiding both signal saturation and excessively weak signals. The selected gradient spans approximately two orders of magnitude around the anticipated *KD* value, consistent with the fitting requirements for a 1:1 Langmuir binding kinetics model. Concentrations below 1 μM yielded signals too weak for reliable detection, whereas those exceeding 100 μM introduced minor nonspecific interference that could compromise the fitting accuracy. As shown in Figure 8B, an increase in the saccharin concentration corresponded with an increase in the relative response (RU) value, reflecting a direct proportionality between the intensity of the binding event and the concentration of saccharin. Furthermore, the binding curve in Figure 8B illustrates the relative response value at equilibrium when saccharin binds to MAPK3. To obtain kinetic parameters, the sensorgram data across all concentrations were globally fitted via a 1:1 Langmuir binding model. The fitting was performed with Biacore Insight evaluation software, from which the *ka*, *kd*, and *KD* were derived. The goodness of fit, assessed by the chi‐square (χ^2^) value, was 1.21 RU^2^, which is below the commonly accepted threshold of 10 RU^2^, indicating a reliable fit and robust derived parameters. SPR analysis revealed that the interaction had an association rate constant (Ka) of 1.27 × 10^3^ 1/Ms and exhibited a dissociation rate constant (Kd) of 5.73 × 10^−2^ 1/s. By performing a nonlinear regression analysis, we calculated the *KD* between saccharin and MAPK3 to be 45.0 μM, indicating a high binding affinity between the two compounds due to the low *KD* value. Besides, the theoretical Rmax value of approximately 44 RU was calculated based on the net immobilization level of the protein, the molecular weights of the analyte and protein, and the number of binding sites obtained from computational simulation studies. These results are consistent with the binding affinity predicted by our *in silico* simulations, thereby validating the reliability of the interaction between saccharin and MAPK3.

## 4 Discussion

This study provides the first integrated evidence linking saccharin to the pathogenesis of EoE. By combining bioinformational analysis, network toxicology, and machine learning, we precisely identified 6 candidate genes: CPS1, HS3ST1, HS3ST4, IL1RL1, MAPK3, and WNT3. Molecular docking and dynamics simulations revealed strong and stable binding between saccharin and these proteins. As established in the literature, MAPK3 plays an indispensable role in inflammatory responses, Th2 immunity, and epithelial barrier function, underscoring its definitive biological significance (Kaymak et al. 2023). We ultimately selected MAPK3 for SPR analysis. SPR analysis confirmed the binding between saccharin and the MAPK3 protein. Collectively, our findings reveal how saccharin potentially intensifies EoE via modulation of fundamental pathogenic genes.

MAPK3 (also known as ERK1) is a key component of the MAPK signaling pathway and plays a significant role in cell proliferation, differentiation, survival, and inflammatory responses (Liu et al. 2018). During inflammation, MAPK3 can be activated by cytokines and inflammatory mediators, which subsequently regulate the expression of downstream genes and promote the recruitment, activation, and release of inflammatory factors by inflammatory cells (Huang et al. 2025). Research has demonstrated the involvement of MAPK3 in regulating the production of cytokines, such as IL‐4, IL‐5, and IL‐13 (Sun et al. 2020). These cytokines are central to EoE pathogenesis and actively promote eosinophil recruitment and activation, which leads to esophageal mucosal inflammation and tissue damage (Underwood et al. 2022). Furthermore, MAPK3 participates in regulating epithelial cell function, impacting epithelial barrier integrity; this disruption significantly promotes the pathological progression of EoE (Kaymak et al. 2023). These findings suggest that MAPK3 may play an important role in the development of EoE. In fact, studies have shown that MAPK3 has the potential to be a therapeutic target for EoE (Maskey et al. 2022). Therefore, saccharin targeting of MAPK3 is highly biologically rational. The fundamental role of MAPK3 in EoE provides a solid theoretical foundation for understanding how saccharin exacerbates disease by targeting this kinase.

Dietary factors play a crucial role in EoE onset, with the intake of specific foods closely linked to disease risk (Hiremath 2024). Notably, common food allergens in EoE—milk, eggs, wheat, and soybeans—harbor protein epitopes capable of deceiving the immune system. When mistakenly identified as foreign threats, these proteins trigger defensive cascades, inflaming the esophageal mucosa and provoking eosinophil infiltration (Crawford and Wright 2025). Building critically upon this foundation, our research demonstrated that saccharin may similarly promote EoE development by modulating immune and inflammatory responses. The findings provide critical insights for artificial sweetener safety assessments, highlighting the critical need to thoroughly evaluate their potential impacts on immune function and inflammatory responses in food additive evaluations.

Although this investigation revealed possible mechanisms of saccharin activity in EoE through an integrated multidisciplinary methodology, several limitations require attention. First, while our SPR assays conclusively demonstrated the direct binding between saccharin and MAPK3, the interactions with other promising core targets remain computationally predictable and require experimental validation. Second, the functional implications of these interactions, particularly whether saccharin binding indeed activates the MAPK signaling pathway and drives the subsequent inflammatory cascade in vivo, are unclear. Furthermore, the translational relevance of our findings could be strengthened by future pharmacokinetic studies.

On the basis of the current findings and limitations, future work should prioritize: experimentally validating the binding of saccharin with other core predicted targets; conducting in‐depth investigations in cellular and animal models to explore the functional impact of saccharin binding to MAPK3 on MAPK signaling pathway activation and downstream inflammatory responses; and performing relevant pharmacokinetic studies to evaluate the relationship between saccharin exposure levels and its biological effects in vivo. Despite these limitations, our work establishes a foundational framework implicating saccharin in EoE pathogenesis, highlighting the need to consider specific dietary components in disease management and future dietary advice for susceptible individuals.

## 5 Conclusion

We demonstrated that saccharin might exacerbate EoE by directly targeting the key pathogenic protein MAPK3 and activating pro‐inflammatory signaling pathways. These findings reveal an unrecognized dietary risk factor for EoE and necessitate a critical re‐evaluation of artificial sweetener safety for susceptible individuals.

## Author Contributions

Yuan Yang and Tao Guo: Conceptualization, methodology, investigation, formal analysis, and writing‐original draft. Peiyuan Li: Validation, data curation, and software. Kangle Gao: Resources and visualization. Xufeng Ning: Investigation and methodology. Lingshan Zhou: Supervision, project administration, and writing‐review and editing. Weiwei Zhou: Funding acquisition, supervision, and writing‐review and editing. Bin Zeng: Funding acquisition, conceptualization, supervision, resources, and writing‐review and editing. All authors have read and approved the final manuscript.

## Funding

This work was supported by the Hunan Provincial Health Commission Scientific Research Project (20233462), Hunan Provincial Department of Education Scientific Research Project (23A0347), and the Natural Science Foundation of Hunan Province (No. 2025JJ81049 and 2025JJ90143).

## Conflicts of Interest

The authors declare no conflicts of interest.

## Supporting information


**Appendix S1:** fsn371409‐sup‐0001‐AppendixS1.docx.

## Data Availability

The data that support the findings of this study are available from the corresponding author upon reasonable request.
