# Supplementary material for: Saccharin Aggravates Eosinophilic Esophagitis via MAPK3 Interaction: A Network Toxicology and Machine Learning Study With SPR Analysis
Source: Food Sci Nutr. 2026 Jan 7;14(1):e71409. doi: 10.1002/fsn3.71409 (PMC12778444; doi:10.1002/fsn3.71409)
Supplement: Supplementary file 1 — Appendix S1: fsn371409‐sup‐0001‐AppendixS1.docx. [file FSN3-14-e71409-s001.docx]

CPS1-6uel_Saccharin,-6.583

CPS1-6uel_2Saccharin,-6.584

CPS1-6uel_3Saccharin,-6.587

HS3ST1-1zrh_Saccharin,-6.716

HS3ST1-1zrh_2Saccharin,-6.705

HS3ST1-1zrh_3Saccharin,-6.707

HS3ST4-Q9Y661_Saccharin,-6.264

HS3ST4-Q9Y661_2Saccharin,-6.249

HS3ST4-Q9Y661_3Saccharin,-6.264

IL1RL1-4kc3_Saccharin,-4.808

IL1RL1-4kc3_2Saccharin,-4.815

IL1RL1-4kc3_3Saccharin,-4.812

MAPK3_4qtb_0_Saccharin,-6.659

MAPK3_4qtb_0_2Saccharin,-6.686

MAPK3_4qtb_0_3Saccharin,-6.652

WNT3-6ahy_Saccharin,-6.07

WNT3-6ahy_2Saccharin,-6.086

WNT3-6ahy_3Saccharin,-6.068
